# “Focus more on what’s right instead of what’s wrong:” research priorities identified by a sample of transgender and gender diverse community health center patients

**DOI:** 10.1186/s12889-022-14139-z

**Published:** 2022-09-14

**Authors:** Merrily LeBlanc, Asa Radix, Lauren Sava, Alexander B. Harris, Andrew Asquith, Dana J. Pardee, Sari L. Reisner

**Affiliations:** 1 The Fenway Institute, Fenway Health, 1340 Boylston Street, 8th Floor, Boston, MA 02215 USA; 2 Callen-Lorde Community Health Center, New York, NY USA; 3 Brigham and Women’s Hospital, Boston, MA USA; 4 Harvard Medical School, Boston, MA USA; 5 Harvard School of Public Health, Boston, MA USA

**Keywords:** Transgender; public health research, Social influences, Focus group, Patient centered outcomes research

## Abstract

**Background:**

Transgender and gender diverse (TGD) individuals disproportionately experience disparate health outcomes compared to their cisgender peers. This study aimed to collect qualitative data from a sample of TGD community health center patients on health research priorities to inform future TGD-centered research in the field of TGD health.

**Methods:**

Between September–November of 2018, four focus groups (two groups in Boston MA, two in New York NY; *n* = 28 individuals) were held to evaluate community-identified TGD health research priorities with a sample of patients from two community health centers. Thematic analyses were conducted and restricted to social factors impacting health. Findings were incorporated into the development of The LEGACY Project, a longitudinal cohort of TGD patients, assessing the impact of gender-affirming care on health outcomes.

**Results:**

Cross-cutting themes about TGD research priorities pertaining to social factors and health included: (1) Embodiment: understanding and investigating the complex and intersectional lived experiences of TGD individuals; (2) Social determinants of health: the impact of structural and interpersonal stigma on TGD health; and (3) Resiliency and health promoting factors: the need to expand public health research beyond disparities to assess resiliency and health promotion in TGD communities.

**Conclusions:**

Participants identified investigating the impact of social influences on health as a research priority for TGD patients. Recalibrating field norms from individual researcher priorities to TGD population-driven research will help ensure investigators address topics that may otherwise be missed or overlooked and may optimize the reach and impact of research in TGD health.

**Supplementary Information:**

The online version contains supplementary material available at 10.1186/s12889-022-14139-z.

## Background

Transgender and gender diverse (TGD) people are individuals whose gender identity differs from their sex assigned at birth [1]. While research demonstrates that TGD communities are subject to disparate health outcomes compared to their cisgender peers, health research regarding the TGD community remains limited and researchers rarely center TGD voices and perspectives in their projects [2]. Conducting TGD research without the inclusion of TGD community input can contribute to disenfranchising the population, ignoring community needs, and causing investigators to miss key topics most influential to TGD population health.

The Patient-Centered Outcomes Research (PCOR) framework prioritizes engagement of patients and other healthcare stakeholders throughout every stage of the research process in order to identify and focus on research areas most meaningful and impactful to patients [3–5]. This framework conceptualizes patient engagement practices on a continuum which can take multiple forms, such as consultation, advisory boards, focus groups, or shared leadership as investigators. Patients are viewed as key partners who bring invaluable lived experiences and personal expertise to the research enterprise. The PCOR framework has been used to guide research investigations in populations burdened by or vulnerable to health and healthcare disparities across multiple physical and mental health conditions. Its principles aim to advance health equity and to enhance research that focuses on health promotion and disease prevention by addressing external drivers of health from one’s social environment [6].

Applying PCOR principles to conduct TGD health research can foster inclusivity of trans voices and perspectives in the research process while also contextualizing the relevance of research that will ultimately advance scientific knowledge [6, 7]. Bringing research priorities from trans voices to the forefront allows researchers to explore gaps and unique topics regarding health and well-being that might not otherwise be identified [8]. Engaging TGD voices in formulating public health research agendas is imperative to optimize the impact of scientific work, including interventions that can address health inequities.

Current research and literature regarding TGD health and public health consist of topics including gender-affirming hormones and surgical procedures, mental health, HIV prevention and treatment, and sexual health [9]. Clinical trials involving trans people are most often in the HIV domain, with less investigation of other health burdens faced by TGD patients [10]. Research studies on topics linked to social determinants of health have been on the rise only recently, suggesting the need for additional studies about barriers to employment, housing, education and legal protections, as well as discrimination regarding culture, race, and ethnicity [9, 11]. To date, TGD health and public health peer-reviewed research topics and priorities are rarely explicitly described as being from TGD patient voices, thus providing a future direction for scientific research and publishing.

The Four Corners: Trans & Nonbinary Health Research Advisory Network recently published a report on health research priorities of Transgender and Non-Binary people. Data were collected from focus groups with TGD people, with the inclusion of Black, Indigenous, and Other People of Color (BIPOC) and TGD people with disabilities, seeking to obtain research priorities regarding health and healthcare [2]. This report, which centers TGD voices, substantiates the importance of communities contributing to the formulation of research priorities and special topics. Centering TGD patient voices is essential to determine whether current TGD health research priorities encompass the actual needs of TGD patients [12]. Consistent with a PCOR framework, assessment of TGD health research priorities requires ongoing input and consultation from TGD communities in order to identify new research opportunities and topics meaningful to TGD individuals.

This study aimed to collect information directly from TGD patients to identify gaps in current research topics, missed research opportunities, and new topics meaningful to TGD individuals which significantly impact health outcomes and life experiences. This information was initially collected to inform the design of The LEGACY Project, a longitudinal cohort study investigating how gender-affirming care impacts TGD health.

## Methods

### Participants and procedures

The LEGACY Project is a multisite longitudinal cohort of TGD primary care community health center patients [13]. To inform the development of the cohort, we collected input directly from TGD individuals to assess community-driven health research priorities and topics. Four focus groups (FG) were held between September and November of 2018 to evaluate community-identified transgender health research priorities and perspectives within a sample of patients from two federally qualified community health centers, Callen-Lorde Community Health Center in New York, NY and Fenway Health in Boston, MA (*n* = 28 across all four focus groups). Both community health centers have expertise in providing competent care and health services to LGBTQ populations [14]. Findings from the FGs were incorporated into the development of The LEGACY Project, the overarching aim of which is to assess the impact of gender affirming care on health outcomes.

Focus group participants were recruited through in-clinic print flyers and electronic advertisements on social media platforms. To be eligible for the focus groups, participants were required to be primary care patients of either health center and defined as having had at least one medical appointment within the prior year. Other eligibility requirements included (a) being ages 18 years or older, (b) having a gender identity differing from their sex assigned at birth, and (c) the ability to read, speak and understand English.

Prior to participation, individuals provided verbal consent over the phone and completed a brief demographic survey which included age, gender identity, sex assigned at birth, race, ethnicity, and geographic location. The focus groups were facilitated by TGD study staff, led by a primary facilitator and a supporting facilitator [15] FG discussions were held in person, lasted 90 minutes, and participants were compensated with a $25 gift card upon completion. The Fenway Institute Institutional Review Board approved all study procedures.

### Data collection: community advisory board and focus group discussion guide

A Community Advisory Board (CAB) was assembled and comprised of 7 TGD community members, identifying as transgender or nonbinary, to provide community centered guidance on the study design, data collection methods, FG guides, and corroborate research findings of the LEGACY project [13]. This guidance led to the development of a semi-structured FG discussion guide which explored important topics and research priorities among participants, gaps in current research, and perspectives regarding top priorities in TGD health research (See Additional file 1 for FG guide). For the purposes of this study, analyses of TGD participant-identified research priorities and special topics were restricted to social factors impacting health, an important dimension of the PCOR framework requiring exploration for TGD patient-centered research [5].

### Data analysis

All four focus groups were audio recorded, and then transcribed verbatim by a professional transcription service. Thematic analyses were conducted by two independent analysts applying a constant comparison method [16]. Two staff analysts used Dedoose 8.3.17 software to apply initial thematic codes to a subset of each of the four transcripts. A codebook, informed by the focus group guide, was developed using an inductive approach, and then finalized through an iterative process. Analysts compared and resolved discrepancies of thematic codes through sharing notes and memos. Each of the four transcripts were then re-coded using the finalized list of codes to guarantee consistency. The CAB provided a final review of study themes and findings, which were then cross-checked with study investigators and other TGD community members. Quantitative data from the brief demographic survey was analyzed in Excel.

### Reflexivity

Reflexivity calls on the research team to take part in the acknowledgment and analysis of one’s intersecting social positions that impact decisions and interpretations of data collection and analysis throughout the research process [17]. This approach can be used to disrupt and minimize harmful power dynamics, particularly between community members and researchers. The research team was diverse in sexual orientation (e.g., queer, bisexual), gender identity (e.g., TGD, cisgender woman), and racial/ethnic background (e.g., Black Afro-Caribbean, White) and had background in public health and commitment to LGBTQ health equity. The focus group facilitators were TGD study staff, who self-identified as White TGD men (2), a White trans masculine genderqueer person (1), and a Black Afro-Caribbean woman of trans experience (1). The two independent analysts with a background in qualitative research self-identified as a White trans masculine genderqueer person and a White cis woman. After the themes were finalized, the two analysts shared the initial list with three other research team members (a White bisexual transgender man, a White transman, and a White cisgender queer woman, all experienced in qualitative work), who corroborated the themes. Each member’s experiences and positionality have influenced members thinking, perspective taking, and approach to the study, which could have a consequential impact on study participants and results.

## Results

A total of 28 individuals participated among the four focus groups. Participants were a mean age of 33.9 years (SD 12.3; Range 18–66). Participants varied in gender identity, with 10 (35.7%) participants identifying as transgender female, 8 (28.6%) as transgender male, 3 (10.7%) as nonbinary, 4 (14.3%) as male, and 3 (10.7%) as female. 12 (42.9%) were assigned male at birth, and 16 (57.1%) were assigned female at birth. 5 (17.9%) were Black, 12 (42.9%) were white, 3 (10.7%) were Asian, 5 (17.9%) were multi-racial, 3 (10.7%) were another race. In terms of ethnicity, 8 (28.6%) participants were Latinx and/or Hispanic and 20 (71.4%) were not Latinx or Hispanic. Of the twenty-eight participants, 11 (39.3%) participated in Boston, and 17 (60.7%) participated in New York City (Table 1).

**Table 1:** Descriptive characteristics of transgender and gender diverse community health center participants

Characteristic	n	%
Gender Identity		
Male	4	14.3
Female	3	10.7
Transgender Man/Male	8	28.6
Transgender Woman/Female	10	35.7
Nonbinary	3	10.7
Sex Assigned at Birth		
Male Assigned at Birth	12	42.9
Female Assigned at Birth	16	57.1
Race		
White	12	42.9
Black	5	17.9
Asian	3	10.7
Another Race	3	10.7
Multiracial	5	17.9
Ethnicity		
Latinx/Hispanic	8	28.6
Not Latinx/Hispanic	16	57.1
Geographic Location		
Boston, MA	11	39.3
New York, NY	17	60.7

*N *= 28*.* Participants were on average 33.9 years old (SD 12.3), and ranged from 18 to 66 years old

**Fig. 1:**
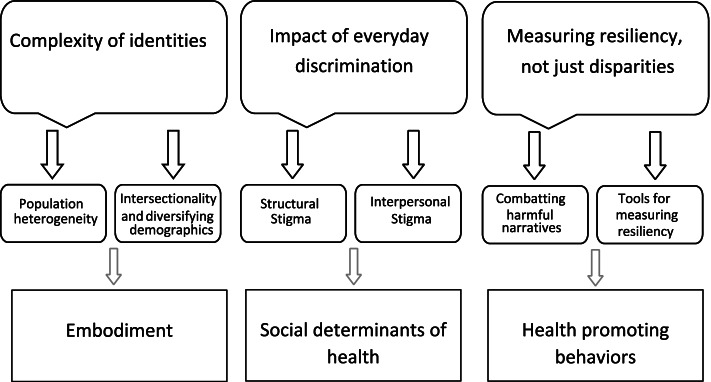
Research priorities of transgender and gender diverse community health center patients

Three research priority areas were identified in FG discussions pertaining to social factors impacting health: (1) embodiment: understanding the complexity of identities, (2) the impact of social determinants of health: structural and interpersonal stigma and discrimination on TGD health; and (3) resiliency and health promoting factors: the need to expand public health research beyond disparities to assess resiliency and health promoting factors in TGD communities (Fig. 1).

### Research priority 1: understanding the complexity of identities

#### Population heterogeneity

Throughout the FG discussions, the need to address the complexity of identities within TGD public health research was repeatedly raised. Participants indicated the need to include and represent a multitude of identities within TGD health research. A participant from New York explained that public health data must be inclusive of the gender identity spectrum to better reflect lived experiences in the context of health and wellbeing:

“[it is important to have] data that actually represent the lived experience. So that means our identities. Right? So, nonbinary people, trans men, trans women, gender nonconforming, like actually that data actually stating those things” (New York Focus Group).

Similarly, a Boston participant expressed the need for “accessibility of the information that there’s not one specific way to be trans” (Boston Focus Group).

TGD participants urged public health investigators to diversify the study samples and increase understanding of the complexity of identities and representation of various identities.

Participants also spoke about the impact of cultural shifts and changes in language as a contributing factor as to why it is important to recognize complexity of TGD identities in research. One Boston participant spoke on the need to assess cultural shifts and changes in language in terms of representation in research:

“there’s some research on people who transition from one gender to the other but all the nonbinary and people in the middle that there’s not a whole lot really known about that. And all that language is still being developed and really those sorts of definitions came about because of the youth in the last like 10 years” (Boston Focus Group).

TGD participants wanted investigators to be familiar and responsive to the reality of varying identities and open to cultural shifts and changes in language, in order to truly contribute to TGD public health research. Allowing trans-specific research to be inclusive of non-binary identities was highlighted as both crucial and timely.

#### Intersectionality and diversifying demographics

TGD focus group participants reported that public health researchers must design their recruitment strategies to include variation in geographic location, income, race/ethnicity, disability, and immigration status, as well as other characteristics. Participants discussed that being intentional to recruit a diverse sample defined by many complex layers of lived experiences and identities would allow the research to be a more representative contribution to the literature. A participant from Boston highlights this need locally:

"Well, you’d want to be able to get to multiple neighborhoods. I mean, if you’re only looking in Cambridge and Fenway [historically predominantly higher income and, white areas of Boston], then you’re missing Mattapan and Dorchester [historically predominantly lower income and Black areas of Boston]. You have to -- it’s got to be a -- For it to be a study that’s got meaning, you can’t be drawing from one strata, whether that means income strata, or one skin color… you’ve got to try to hit all those different rainbow colors” (Boston Focus Group) [15].

The Boston participant offers that those most likely to be missed in research may be the most important to involve. Conversations from the focus groups conclude that it is important for researchers “to outreach,” “get more representative samples,” and “get everyone’s voice involved” (New York Focus Group).

### Research priority 2: the impact of structural and interpersonal stigma and discrimination on health

#### Structural stigma

A research priority drawn from both Boston and New York FGs was investigating the ways in which TGD individuals and communities experience everyday discrimination. Discussions in FGs about the importance of addressing everyday discrimination highlighted the need for TGD health research to include considerations of structural stigma. Examples of everyday discrimination mentioned by FG participants include limited access to housing, employment, health insurance and supportive health care.

Participants expressed a need to incorporate one’s living arrangements, including housing and neighborhoods, when investigating TGD health outcomes. One participant from the Boston FGs spoke about the potential impact of unsafe living arrangements on their health and wellbeing by sharing a personal experience:

“I think it’s really important where you live, and who you live with, and who your management is, and, you know, who your neighbors are, what kind of neighborhood do you live in. I think it’s really important that -- it really have an effect on your mental health, which has an effect on your physical health, you’re living in an environment, if you come here, [Fenway Health] like -- I go to a group once a month and I went to the group last month and I was like -- and I haven’t been there in like five years, and I was like, ‘I feel really safe today.’ And sometimes I forget how unsafe I feel” (Boston Focus Group).

In addition to facing structural barriers such as safe housing, another participant introduced barriers in navigating employment as a contributing factor to TGD quality of life and health not often considered as a TGD health research topic:

“What I really want to discuss is, being trans while employed -- what that is -- how to -- how to navigate being a transgender employee. Because I’ve found nothing but difficulty” (New York Focus Group).

Additionally, TGD participants shared that they had faced further structural stigma-related barriers with health insurance. One participant offered a personal example in utilizing their Medicaid policy and noted a discrepancy in communication due to their name change.

“[an] experience dealing with insurance is that they’re not sensitive to the name change because you can go by the name you want but that doesn’t mean legally that your name has changed yet. And then when your medical insurance like Medicaid wants to give you a phone call they call you by that name. And it’s like no” (New York Focus Group).

#### Interpersonal stigma

The stress experienced from interpersonal social stigma and its effect on health was identified as a main research priority in the FGs. Participants shared that their experiences of street harassment and harmful interactions with others were stressors that impact their health and well-being. One participant shared their experience interacting with a housing manager who exhibited discriminatory behavior towards them. The resulting stress from this interpersonal interaction influenced the participant to remain home rather than attending their health appointment. A fellow participant agreed that discrimination is the root of the problem:

- PARTICIPANT 2: “But he was on a track to totally try to get me kicked out of the building and it’s because he’s -- you know, he’s a racist, homophobic, transphobic kind of person. But that kind of stress -- so, that stress, honestly, I haven’t been to the Fenway [Health Center] -- I’ve cancelled all my appointments of the whole month. I haven’t been there in a month because I’m staying in the house. So, you know, if it weren’t for this group, that’s where I’d be, just locked in my house.”
- PARTICIPANT 4: I think what you’re getting at too is discrimination. That’s discrimination.
- PARTICIPANT 2: Right, and I think that really has an effect on your health.
- PARTICIPANT 4: Absolutely.” (Boston Focus Group).

This conversation between the two participants regarding experiences of everyday discrimination from others also resonated strongly with other participants. One Boston participant shared the need to evaluate interpersonal discrimination more thoroughly in order to understand how it impacts various aspects of life including health and mental health:

“And that’s something that I don’t think we’re ever asked on those surveys that we get, and that really -- I experienced discrimination getting my name changed, which is, like, unheard of in Boston, and a lot of people have not believed me when I’ve talked about it, and my primary care and my therapist here are the only ones that are really on my side with that. And that’s -- you know -- discrimination because of who we are happens all the damn time. But we’re not really given the platform or the opportunity to have our voices heard about that, and I don’t know if maybe that’s something -- because, like you say, it does affect our mental and physical health. If that’s something that could really be added, and different levels of that and maybe how it intersects with other aspects of our lives” (Boston Focus Group).

This participant expressed the nuances and challenges associated with disclosing experiences of discrimination, even in a seemingly affirming environment. This sentiment was echoed from other participants who spoke about the need for investigators to consider the impact of everyday discrimination including both interpersonal stigma and structural stigma, when conducting public health research unique to the TGD communities.

### Research priority 3: measuring resiliency and not just disparities

#### Combatting harmful narratives

Participants in both Boston and New York expressed the need to move beyond studying and addressing disparities within the community and to include research questions regarding resilience. A Boston Participant expressed:

“Yeah, it would be nice to do more studies on the resilience of trans folks and -- because we know that there -- and also to look at things from a systemic, you know, things of like minority stress and how that affects us but then also our resilience and how we power through despite the incredible amounts of oppression we face. That would be, like, big systems studies would be cool” (Boston Focus Group).

TGD individuals face a high level of adversity and are subject to oppressive systemic structures and yet subsequently embody resilience. Participants felt that continuing to only evaluate disparities perpetuates harmful narratives about TGD people; measuring resilience can aide in the effort to combat these harmful narratives. A New York participant shared that:

“every time it’s related to trans all I hear is it’s related to HIV and STDs and STIs. And sexually whatever whatever. And I would just like to understand like why is that because it just feel like you put a label on me and that all of us seem to carry around diseases with each other but that’s not the case” (New York Focus Group).

In contrast to disparity research, adding resiliency research and other topics in which TGD people experienced success or have seen benefits and improvements was thought to help minimize stigmatizing narratives associated with TGD identities. FG discussions identified that conducting and disseminating research on positive health outcomes would be a desirable research priority. New York participants shared:

“Want to hear more success stories for sure. Focus more on what’s right instead of what’s wrong for a change. I like that,” (New York Focus Group).

and

“I think also sometimes we focus on like what’s wrong. Like how prevalent is this problem in this community. But I want to see more research on what helps us and things to ask for from institutions and be like, “This is proven to help trans people” (New York Focus Group).

FG participants highlighted several topics, including recovery from alcohol and/or drug abuse and sexual health promotion as examples of encouraging resilience research topics that are often overlooked or missed.

#### A tool for measuring resiliency

Participants described experiences where focusing only on disparities or prevalent adverse health issues seen in TGD communities may leave TGD patients feeling the weight of these issues. For example, one Boston participant spoke about their past experience filling out questionnaires and answering questions at the doctor’s office:

“you walk away with, I don’t know, ‘should I be feeling worse about myself?’ ” (Boston Focus Group).

Focus group participants suggested that there should be a way to formally evaluate positive aspects of TGD life experiences rather than only assess the challenging aspects. One participant spoke on this experience and offered a suggestion:

“You know, maybe it’ll be worded like, ‘were you depressed five days out of the last month?’ But nowhere is there anything similar to, ‘were you extremely joyful 14 days out of the last month?’ ” (Boston Focus Group).

The FG discussed the positive impact a measure of resilience as a health promotion tool could add to the TGD healthcare experience. Participants stated that without looking at resilience, investigators will miss capturing the extent and depth of the TGD experience.

## Discussion

In this study, TGD individuals provided input on priorities and topics about social influences on health for future research. The top research priorities and special topics identified by this sample of TGD community health center patients coalesced around the overarching and overlapping themes of the complexities of identity, the impact of structural and interpersonal stigma and discrimination on health and measuring resiliency and not just disparities. The complexity of identities of TGD individuals can be uniquely understood as “embodiment,” a construct that identifies an individual body as simultaneously biological and rooted in social contexts, highlighting the need for investigators to consider and include the complexity and representation of heterogenous identities of the transgender and gender diverse population in research [18]. Social Determinants of Health look beyond medically-related determinants to include evaluating social conditions unique to TGD people and experiences of structural and interpersonal stigma and discrimination that may impact health [19]. Investigating health promoting factors, such as measuring resilience and not just disparities, will combat harmful social narratives that reproduce social stigma and better evaluate the lived experiences of TGD individuals. Many of the research priorities and special topics identified by participants overlap, which emphasizes the importance of social influences on health as told by TGD participants. These three themes have implications for interventions, policy, clinical practice, and research relevant to TGD health outcomes. Future pursuits in TGD health research should include evaluation of social influences on health in addition to TGD community member input and involvement in all aspects of the study design.

### The complexity of identities: embodiment

The concept of embodiment reflects that we are simultaneously social and biological organisms whose lived experiences cannot be separated from social context [18]. As referenced by participants, social contexts as well as language and definitions surrounding TGD identities have evolved to counter social expectations of gender identity. Further, understanding the complexity of identities in the context of embodiment decentralizes an over-medicalization and bio-essentialist perspective of TGD patients’ health status and gives weight and attention to social contexts. Cultural shifts in TGD communities must be considered in research questions attending to the causes of health disparities.

Participants expressed the need for investigators to consider the heterogeneity within the TGD community; namely, as one participant mentioned, there is “not one specific way to be trans” (Boston Focus Group). By expanding gender identity categories beyond the binary and including non-binary, genderfluid, and gender diverse identities in research, investigators would better represent population characteristics and create visibility of marginalized identities within the TGD community. Social conceptions of gender identity typically rely on female and male binary categories, but public awareness of nonbinary identities has increased over time. For example, in 2014, the social media outlet, Facebook, increased their gender identity categories from only male or female to over 50 options for users to select [20]. Participants in this sample expressed how shifts in culture play a role in developing language regarding complex TGD identities. Emergence of these diverse identities in the public discourse impacts both the individual and social context, making gender an important health-related factor.

This sample of TGD participants urged investigators to expand representations of identities in TGD health research. TGD participants highlighted the importance of considering race/ethnicity, sexual orientation, age, socioeconomic status, and immigrant status among TGD populations and further suggested expanding beyond these demographics. Expanding representations of TGD individuals in health research, such as those with non-binary identities and those whose primary language is not English, could contribute to more accurate evaluations and reflections of the lived experience of TGD communities and in turn, generate richer data. In doing so, researchers will achieve a more diverse sample and gain an opportunity to use an intersectional lens when establishing research priorities. Intersectionality is a framework that acknowledges the ways in which multiple forms of oppression exist at the intersection of marginalized identities [21, 22]. Initially conceptualized in feminist theory, an intersectional lens can be extended to TGD population health as it has developed to include intersections between multiple social differences and identities [21, 22]. Using an intersectional framework implores researchers to expand study populations thoughtfully and intentionally beyond the demographic of “trans.” Participants emphasized inclusion of individuals traditionally disenfranchised from research participation to create a more representative sample and ensure the diverse expression of TGD voices and experiences. One example from the FGs included an effort to recruit research participants from hard to reach or seldom heard TGD communities from varied neighborhoods. Using an intersectional lens in conducting TGD health research will facilitate a greater focus on the impact of embodied experiences of multiple oppressions and the complexity of TGD identities. Future academic-community partnerships to devise innovative sampling methods for TGD health research is needed.

### The impact of structural and interpersonal stigma and discrimination on health: social determinants of health

The social determinants of health framework is a theme reflective of TGD participants’ consensus that the impact of everyday discrimination on health is an important research priority. Social determinants of health (SDOH) are the conditions in which people are born into and continue to exist in throughout their lives that play a role in health and well-being [19]. TGD FG findings underscore that investigators should consider SDOH-related stress exposures, such as discrimination, living, and working environments, as a contributor to TGD health status when developing and formulating research agendas.

The Healthy People 2020 and 2030 reports aim to improve health and safety in neighborhoods where people live, work, learn and play. Significant health risks in unsafe neighborhoods include increased crime rates, exposure to violence, detrimental environmental impacts such as pollution and access to clean water, and limited access to healthy food [19, 23]. TGD participants described the role of neighborhoods and living environments as having a profound impact on their health and well-being and identified this as a priority area for investigators. One Boston participant who spoke about the need to diversify demographics in trans-related health research also discussed the variability of living conditions and health outcomes that occur in across different neighborhoods. For example, in Boston, historically, the lower-income areas of Mattapan and Dorchester experience poorer health and lower life expectancy than in the higher-income areas of Cambridge and the Fenway [24]. Do TGD people living in lower-income areas, compared to higher-income locales, fare worse than cisgender individuals living in those same neighborhoods? The answer to this, and many other research questions about neighborhoods and TGD health, remain unknown and necessitate future investigation.

Examples of everyday discrimination described by FG participants can be defined as structural stigma: the cultural, institutional, and societal-level conditions that contribute to limited opportunities and negatively impact one’s well-being [1, 25]. Study participants described how the daily occurrence of structural stigma involved in navigating employment and health insurance creates chronic stress for TGD populations. Prior research from the 2015 U.S. Transgender Survey (USTS) reported that 30% of TGD respondents experienced some form of mistreatment in employment, including being fired or denied a promotion due to their gender identity or expression [26]. High rates of unemployment alongside mistreatment in work environments contribute to experiences of social stress and structural stigma for TGD people. Further, employment barriers were identified to contribute to limited economic stability, inconsistent health insurance coverage, and disruptions in needed healthcare and mental healthcare services for TGD people. TGD patients reported facing structural stigma in commercial insurance as well as in other insurance types not dependent on employment status such as Medicaid and Medicare. One FG participant described an experience with structural discrimination in utilizing Medicaid and the lag time and discrepancy due to the process of legal name changes. These qualitative findings are corroborated prior quantitative research demonstrating that as many as one in four TGD respondents have experienced a problem with insurance due to their gender identity, including denial of routine care and care related to their transition, and over half have been denied coverage from transition related surgeries [26]. Additional research is needed to not only further understand the health-related impacts of navigating employment and health insurance issues, but also to intervene to improve these structural barriers for TGD people.

In addition to structural stigma, FG participants highlighted the need to consider the effects of interpersonal stigma, both the implicit and explicit experiences of discrimination between stigmatized individuals and non-stigmatized individuals [1]. Participants reported that social stigma and discrimination was present in their everyday interactions with healthcare center staff and other health professionals, as well as peers, friends, and family. Participants also expressed experiences of street harassment and discrimination in other public spaces such as the workplace and living environments. The stress of social stigma and discrimination was described as inhibiting some participants from engaging in a social life or attending their health appointments. Exposure to everyday discrimination is considered a social determinant of health by the Healthy People Report of 2020, particularly prevalent in TGD communities, and related to their stigmatized identity [11]. FG discussions pertaining to the unique impact of everyday discrimination experienced by TGD participants on health and wellbeing closely align with understandings of social determinants of health for stigmatized identities and should be further built upon in TGD health research. TGD participants’ perspectives highlight the vital need to consider social determinants of health in research, including biomedical research.

### Measuring resiliency and not just disparities: health-promoting factors

This sample of TGD participants identified the need to measure resilience and not just disparities as a resonate research priority. TGD participants further detailed how doing so would combat harmful narratives, and suggested creating a tool for measuring resiliency as a health promotion factor.

Resiliency is the ability to cope and manage stress, often built from experiencing challenging situations [27]. TGD individuals are faced with a high level of adversity and are subject to oppressive systemic structures including social, political, economic, and cultural barriers and yet TGD individuals subsequently embody resilience [27, 28]. FG participants urged investigators to include resiliency research alongside health disparity research in order to highlight resistance to social oppression and combat harmful narratives that reproduce social stigma. Some factors have been shown to promote health among TGD individuals including family support, peer support, community engagement and activism, and spiritual beliefs [1] Understanding health-promoting factors and how they enhance resiliency was identified as a research priority by the FGs and echoed findings in the 2020 Understanding the Well-Being of LGBTQI+ Populations NASEM report. FG participants spoke on the impact that researching topics such as recovery from substance use or sexual health promotion would have on producing counter-narratives. Building upon strategies that TGD communities have used, or currently use, to resist oppression may enhance the opportunity for evidence-based interventions to address health disparities [1].

TGD participants suggested creating a tool to measure resilience among researchers and clinicians to better evaluate and elaborate the depth of TGD health-promoting experiences. FG discussions affirmed that it is vital to measure adverse health outcomes such as depression and anxiety, but emphasized it may be equally important to measure elation, joy, and other positive health outcomes [15] Integrating a strengths-based approach in investigations was highlighted as an important way forward for research to identify and enhance TGD communities’ unique resiliencies while recognizing the stigmatizing social context that the community is embedded in. Learning about a population’s potential, strengths and capabilities is important in countering the focus on adverse health effects [29]. TGD participants voiced the need for a resilience-focused approach in TGD health research – moving beyond the sole focus of studying “sick” people and populations, and moving from a study of surviving to the study of thriving. Both individual and community resilience can act to buffer the negative effects of social stress on health thus playing a crucial role in improving health outcomes [28, 30].

## Limitations

Findings from this study should be considered alongside several limitations. Participants were TGD primary care community health center patients receiving care in urban cities with well-resourced academic medical systems equipped to offer many gender-affirming care services. Therefore, we do not know the extent to which findings can be generalized to TGD community members outside of primary care service or in other health care settings (e.g., hospital clinics) or geographic locales (e.g., rural areas). Further, TGD participants in this study may not reflect or generalize to the clinic populations, given this was a non-probability sample. Future efforts should extend beyond primary care patients from TGD population-specific community health centers. Another limitation was the small sample size of 28 participants; however, thematic saturation was reached as is appropriate in qualitative research. Strengths of the study included the involvement of the Community Advisory Board and self-awareness by the research team of our own positionality in data collection and analysis.

## Conclusion

This study sought to gather insights directly from members of the TGD community about research priorities, needs, and topics meaningful to TGD individuals. The approach of integrating TGD communities reflects patient-centered research practices, a recommendation for conducting TGD health research described by participants within this study. Participants identified multiple cross-cutting themes related to social influences on health and well-being for research. Findings highlight the need for investigators to 1) intentionally represent and investigate the embodiment of complex and heterogenous TGD identities in studying health, 2) consider the role of structural and interpersonal stigma on health, including applying a social determinants of health framework to assess the lived experiences unique to TGD people, and 3) assess the exceptional resiliency encompassed by the TGD community and include health-promoting factors in research.

Given findings from this sample of TGD patients, it is important for research investigators to further explore the social influences on health and well-being. We encourage investigators to include recommendations and insights from TGD patients when creating a health research agenda. From a clinical perspective, TGD health research should look at the biomechanisms of social stress, stigma, and discrimination experienced by TGD individuals. Investigating the impact of structural barriers and social determinants of health may inform social policies related to one’s living environment including housing, employment, and health care. Investigators should remain cognizant of shifts in language and attitudes to ensure research is gender-affirming and culturally responsive when considering the TGD community. As an intervention component, researchers and clinicians should incorporate collective resilience practices of TGD communities to combat harmful narratives, empower patients, and expansively evaluate in-depth the lives and health of TGD patients. Efforts to use a strengths-based approach and resilience frameworks with a health promotion model would be a meaningful direction for future work and considerations.

Recalibrating field norms from individual researcher priorities to TGD population-driven research will help to ensure investigators more effectively address topics that might otherwise be missed or overlooked, and optimize the impact of research within the field of TGD health [8, 31]. Research partnerships co-led with TGD communities can help to ensure that current population health research is meaningful to TGD individuals and communities. Centering TGD people throughout the entire research process by designing equitable partnerships, from design to dissemination, will ensure impactful and relevant research that incorporates health outcomes and life experiences unique to TGD communities (Table 2).

**Table 2:** Key take-aways for research priorities identified by a sample of transgender and gender diverse community health center patients

**Key Take-Aways:**			
*When conducting transgender and gender diverse (TGD) health-related research:* • Recalibrate field norms from individual researcher priorities to TGD population-driven research • Co-lead research partnerships with TGD communities • Center TGD people throughout the entire research process by designing equitable partnerships from design to dissemination			
Clinical Perspectives	Policy	Research	Interventions
Examine the biomechanisms of social stress, stigma, and discrimination experienced by TGD individuals	Investigate the impact of structural barriers and social determinants of health to inform social policies related to one’s living environment including housing, employment, and healthcare	Remain cognizant of shifts in language and attitudes to ensure research is gender-affirming and culturally-responsive when considering the TGD community	Incorporate collective resilience practices of TGD communities to combat harmful narratives and empower patients Validate tools for measuring resiliency to expansively evaluate the lived experiences and health of TGD patients beyond disparities

## Supplementary Information

**Additional file 1.**

## Data Availability

The datasets generated and/or analysed during the current study are available from the corresponding author on reasonable request.
